# Quantification of soil inorganic carbon using sulfamic acid and gas chromatography

**DOI:** 10.1371/journal.pone.0320778

**Published:** 2025-05-05

**Authors:** Christopher Yip, Philip D. Weyman, Kimberly A. Wemmer, Yun-Ya Yang, Anupam Chowdhury, Bjorn A. Traag, Tania Timmermann, Gonzalo Fuenzalida-Meriz

**Affiliations:** Andes Ag, Inc., Alameda, California, United States America; Bristol-Myers Squibb Company, UNITED STATES OF AMERICA

## Abstract

Carbon dioxide is the primary greenhouse gas emitted through human activities, and these emissions impact the carbon cycle for hundreds to thousands of years. As carbon dioxide removal strategies to address this challenge continue to be explored and scaled, faster methodologies with high accuracy and precision are required to support the carbon measurements on which these strategies hinge. Of the many available methods to measure soil inorganic carbon, only a select few satisfy all the following criteria: measure inorganic carbon directly, use standardized equipment, perform the measurement automatically, and operate at high throughput. In this work we present a robust protocol for the sensitive and specific quantification of inorganic carbon from soils using gas chromatography to quantify carbon dioxide evolved from carbonates in soil with sulfamic acid. We demonstrate the precision of this method with purified carbonates, carbonate minerals, biogenic carbonates, and agricultural soil samples. We also demonstrate the accuracy of this method by adding known amounts of calcium carbonate to a variety of soil matrices. We find that sulfamic acid is well suited for carbonate dissolution and is compatible with gas chromatography applications, and we note that the method generates results that are equivalent to the typical methods used in this field. This method is compatible with automation and operation at a greater scale and enables the creation of higher resolution soil inorganic carbon datasets.

## Introduction

Carbon dioxide (CO_2_) is the primary greenhouse gas produced through human activities [1–3] and contributes significantly to global climate change. Limiting temperature increases to 2 °C by 2100 will require both emission reduction and removal of greenhouse gases such as CO_2_ from the atmosphere [4,5]. Deposition of inorganic carbon into crop fields could be an important mechanism for carbon sequestration [6–8]. As such, agricultural land has the potential to be a major contributor to achieving net zero CO_2_ emissions and limiting the further impact of global warming [9], and, therefore, there is an urgent need for frequent and accurate quantification of soil inorganic carbon (SIC) from this setting.

SIC is mainly composed of carbonates (CO_3_^−2^) and bicarbonates (HCO_3_^−^) of calcium (Ca^2+^), magnesium (Mg^2+^), potassium (K^+^), and sodium (Na^+^) [10]. Carbonate minerals exist predominantly as calcite (calcium carbonate, CaCO_3_), dolomite (CaMg(CO_3_)_2_), and other sparingly soluble alkaline-earth carbonates. Calcite is the most stable CaCO_3_ phase and exists in a variety of forms and sizes, ranging from sub-micrometer particles to nodules of 1 cm or greater, to well-formed rhomboids [11]. While carbonates are found in many forms in agricultural soils, total carbonate concentration is often expressed as calcium carbonate equivalents (CCE%) as a percent by mass.

Several methods are commonly used to estimate SIC, including pressure calcimetry (PC), combustion, gravimetric and volumetric methods. Available methods to measure carbonates in soil can be split into two main groups: methods that rely on an acid treatment to turn the carbonate fraction into CO_2_ and methods that rely solely on selective combustion of organic and inorganic carbon. In the later set of methods, a slow temperature ramp during combustion allows measurement of organic carbon at lower temperature before heating to higher temperature for the measurement of the inorganic [11–13].

A wide variety of methods rely on acidification of the soil to convert the carbonates into CO_2_ which can then be quantified by a variety of measurement techniques. Some techniques directly measure the CO_2_ released from the soil sample by acidification of the carbonates and include the gas chromatography (GC) method described in the paper, methods quantifying CO_2_ with infrared (IR) detectors, methods using volumetric determination, and methods that measure pressure using a manometer (i.e., PC) [11,14]. Alternatively, inorganic carbon can be estimated using an indirect method where acidified soil samples are compared with non-acidified counterparts and the difference is assumed to be the inorganic fraction of the soil. Examples include gravimetric analysis of the soil where differences in soil mass before and after acid treatment are compared, as well as methods employing rapid soil combustion where the released CO_2_ is measured by IR and any decrease in acid-treatments is assumed to be the inorganic carbon fraction. All these methods to measure SIC have their own challenges and limitations, as expanded on in the discussion. In addition to having necessary accuracy and precision, one of the biggest concerns is sample throughput. This is especially true given the large number of samples resulting from the need to build out models to accurately predict the flow of carbonates and bicarbonates from agricultural systems and through the carbon cycle.

In order to increase throughput, we aimed to develop a method that is rapid enough to process hundreds of samples per day but is sensitive and reliable enough for precise and accurate readings of carbonates in a complex matrix, such as soil. For operational ease, throughput capacity, precision, and reliability, we chose GC as an instrument to perform this method. Others have previously used GC to detect carbonates in aqueous samples such as environmental water [15] or wastewater [16], enabling monitoring of these chemical species in waterways. Dai et al. demonstrated this technique further with solid substrates, quantifying the amounts of calcium carbonate additive included in paper during production [17]. Finally, Liu et al. determined that the same basic approach using GC functions for more complex substrates as well by looking at the calcium carbonate levels in barite ore [18].

While the available methods for GC provided a good starting point for our method development, they suffered from some critical limitations. First, some of the previously described methods measuring carbonates with GC use hydrochloric acid (HCl) as the acid [16,18]. This should be avoided since the volatility of HCl can damage the metal components of the GC. Other acids such as sulfuric acid have been used with GC to evolve CO_2_ from carbonates [17], but we suspected they would not completely dissolve carbonates in soil [19,20]. Therefore, we used these previous reports as a basis for inspiration to develop the new GC method described in this paper.

The primary goal of this study was to establish a high throughput framework for the robust and precise quantification of SIC from agricultural soils using GC. Potential processes and applications that occur downstream of carbonate dissolution were also considered; the effect of acids on liberating CO_2_ from CaCO_3_ powder, mineral calcite, and biogenic calcite was investigated. Here, we demonstrated the feasibility, accuracy, and high sensitivity of the GC method. We found that sulfamic acid, H_3_NSO_3_, is the superior choice for carbonate dissolution compared to hydrochloric and sulfuric acids due to its non-volatility, lack of reaction precipitates that may coat reactants, and complete digestion of SIC from soil samples. The methodology reported in this study could be modularized and further expanded to other matrices, such as agricultural leachates and porewater in support of carbon measurement, monitoring, reporting, and verification processes.

## Materials and methods

### Chemicals and materials

A standard hydrochloric acid (HCl) solution (1.5 mol/l) was prepared in a 1000 ml beaker by dissolving 250 ml 6 M HCl (Ricca Chemical Company) into distilled water (dH_2_O). A standard sulfuric acid (H_2_SO_4_) solution (2 mol/l) was prepared in a 1000 ml beaker by dissolving 112.3 ml H_2_SO_4_ (purity >95%, Sigma Aldrich) into dH_2_O. A standard sulfamic acid (H_3_NSO_3_) solution (1.5 mol/l) was prepared in a 2000 ml beaker by dissolving 291.27 g sulfamic acid into dH_2_O. Iron (II) chloride tetrahydrate (FeCl_2_ • 4H_2_O, LabChem) was added to all acid solutions to a final percentage of 3% (w/v) to inhibit the release of CO_2_ from organic matter [11,21–23].

### Preparation of samples, standards, and controls

To prepare CaCO_3_ standards for standard curves, 0.5 g silica sand (400 mesh, >99.2% SiO_2_, PMC Supplies, Lake Katrine, NY) was weighed into 22 ml GC headspace vials (Thermo Scientific), adding 2–150 mg CaCO_3_ (Chem-Impex International, >99% purity), and capped tightly (screw caps with blue silicone/clear PTFE, 2 mm-thick septa, Thermo Scientific). Some experiments used larger grain calcite crystals rather than the CaCO_3_ powder used for standards. In these experiments, each GC vial contained 20 mg calcite. Commercially available calcite (Ward’s Scientific) was research grade sourced from Chihuahua, Mexico (>85% purity), and had an average grain size of 3.75 mm.

We selected agricultural soils from the Midwestern region of the United States that vary in their amounts of CCE% to test our GC method (S1 Table 1). For experimental samples, 0.5 g dried, sieved (2 mm, mesh #10), and ground soils were transferred to 22 ml GC headspace vials, and capped (as for controls). For some analyses, CaCO_3_ powder was added to the soils. For this, samples were ground with a mortar and pestle with different levels of CaCO_3_ powder until a near homogeneous mixture was obtained.

Prior to analysis with PC or GC, each sample was injected with 5 ml 1.5 M HCl or 1.5 M H_3_NSO_3_ through the septum with a 22-gauge hypodermic needle. Acids also contained 3% (w/v) FeCl_2_. After acid injection, the samples were gently mixed and incubated at room temperature for 24 hours before measurement. Alternatively, when processing larger numbers of samples, acids were injected using a Hamilton Microlab 600 configured for continuous dispense with 25 ml syringes and a luer lock adapter connected to a 22-gauge needle. For quality control, an internal control was run for every batch of 20 soil sample vials that consisted of 20 mg CaCO_3_, and the analytical run was considered to pass if the measured and expected values did not differ by more than 20%.

All raw data from all GC and PC experiments can be found in S2 Table 2.

### Pressure calcimetry analysis

A modified pressure-calcimeter (PC) method was used to quantify CCE% in soil samples [24,25]. In brief, a pressure transducer (Model 280 G Serta, 0–15 PSI, output 0.08–5.08 VDC from Setra Systems, Inc.), connected with a power supply and a digital multimeter (Digi-Sense compact digital multimeter from Cole-Parmer), was wired in line to monitor the output from the transducer. Tubing connected to a 22-gauge needle with a 0.6 µm filter (Leco Co.) was attached to the transducer. The needle was used to pierce the vial septa prepared as described above, and the pressure resulting from the evolved gases was recorded once the output reading stabilized.

### Gas chromatography analysis

CO_2_ (g) was evolved in sealed, 22 ml glass headspace vials containing either controls or soils after injecting 5 ml acid. The samples were incubated at room temperature for 24 h, and 1 ml of headspace was sampled with a Combi-PAL autosampler system (CTC Analytics) fitted with a 2.5 ml Hamilton gas-tight syringe. Headspace gases were separated on a Thermo Scientific TRACE 1310 Gas Chromatography system fitted with a Restek RT-Q-Bond PLOT column and thermal conductivity detector (Thermo Scientific 1300 IN Series).

The column flow rate was set to 9.8 ml min^−1^ with a split ratio of 10, using helium as a carrier gas. Inlet temperature was 100 °C, and the oven temperature was set to 50 °C for 0.8 min followed by a ramp to 90 °C at a rate of 80 °C min^−1^.

This method measures the CO_2_ released from biogenic carbonates and carbonate minerals, and any bicarbonate and carbonate ions in solution. The areas under the peaks corresponding to CO_2_ and air (primarily N_2_ and O_2_ that were not separated by the column) were determined using Chromeleon 7 software (Thermo Scientific). As described in the Results section, a standard curve for the mass of CaCO_3_ equivalent in a sample was constructed by plotting the ratio of the peak areas for the CO_2_/air peaks as a function of the CaCO_3_ mass in milligrams.

### Formation and harvesting of biogenic calcite

Overnight cultures of *Bacillus subtilis* MP2 were grown in 3 ml B4+ (0.4% yeast extract, 0.5% tryptone, 0.5% glucose, pH 8.2) then spread onto B4+ agar containing 25 mM Ca(OAc)_2_. The cultures were incubated at 30 °C for 5–7 days and monitored for crystal formation using a Leica S9i digital stereo microscope. The plates were flooded with dH_2_O and the biomass and crystals were harvested by scraping the bacterial lawns, collected into 50 ml conical tubes, and pelleted at 2000 xg for 5 minutes. The crystals were separated from the biomass through a sequential series of washes in which the crystals were resuspended, allowed to settle for 5 minutes, and the supernatants were removed by aspiration: (1) dH_2_O, which removed most of the biomass, (2) boiling water, (3) 70% isopropanol (to remove trace organics), and (4) 100% isopropanol. Crystals were dried at 70 °C for 1 hour, then stored at room temperature prior to use.

### Test for acidification of headspace

B4+ agar containing 0.0025% bromophenol blue was sterilized and 2 ml was transferred into 10 ml GC vials (Thermo Scientific) and allowed to solidify. The vials were capped, inverted, and 2 ml acid at equivalent normality (see Chemicals and Materials section for actual molarity of each acid) was slowly injected through the septa into the bottom of the vial such that the liquid was kept away from the agar. For the untreated controls (UTC), 2 ml dH_2_O was carefully injected. Acid vapors from volatile acids, such as HCl, cause the protonation of bromophenol blue resulting in the indicator to transition from blue to yellow. The vials were incubated at 37 °C for 3 days, then photographed.

### Statistical analysis

In experiments in which different levels of CaCO_3_ were added to soils, CCE% in treatments was compared by ANOVA followed by means comparison with Tukey’s all-by-all test using the statistical package JMP software version 17. For heteroscedastic sample groups, Brown-Forsythe and Welch ANOVA test was used with means comparison by Dunnett’s multiple comparisons test using the software package Graphpad Prism version 10.2.3.

The equivalence of the soil CCE% measurements analyzed by the three assays, i.e., (1) PC with HCl, (2) PC with H_3_NSO_3_, and (3) GC with H_3_NSO_3_, was analyzed using two one-sided t-tests (TOST) [26]. In brief, a margin of equivalence (M) is set for the maximum allowable difference in mean CCE (μ_1−2_) reported between any pair of assays. Subsequently, two paired Student t-tests are performed on the difference of soil CCE% values for analyzed samples to reject the null hypotheses that μ_1−2_ <−M and μ_1−2_ > M, with a significance level α. For the current analyses, the TOST equivalence test was performed between each pair of assays with M=1 CCE% and α=0.05 in JMP software version 17.

## Results

### CaCO_3_ dissolution and evolution of CO_2_ with HCl, H_2_SO_4_, or H_3_NSO_3_

Gas chromatography is a powerful technique used for the analysis of volatile compounds. Phase reaction conversion of solid CaCO_3_ to gaseous CO_2_ can be easily achieved following the addition of an acid [27]. To demonstrate this, the concentration of each acid (HCl, H_2_SO_4_, and H_3_NSO_3_) was first adjusted such that the number of protons in each case was equivalent. Next, 5 ml of each acid was injected into 22 ml vials containing 40 mg CaCO_3_ powder resulting in the rapid evolution of CO_2_, evident by sample effervescence.

**Fig 1:**
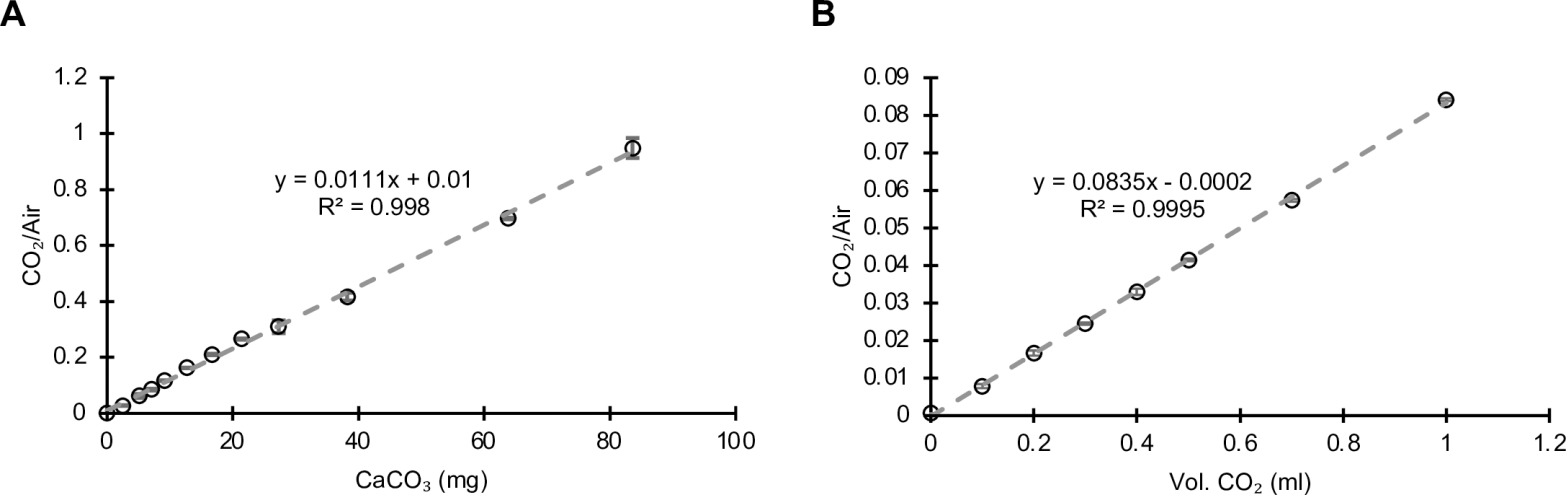
Quantification of a CO_2_-specific signal from atmospheric gases. (A) Standard curve resulting from increasing inputs of CaCO_3_ from 2.5 mg to 83.6 mg per vial. (B) Standard curve using dilutions of CO_2_ in air from 0.1 ml to 1 ml CO_2_ added to 22 ml GC vials. The signal from this amount of CO_2_ corresponds to the equivalent of 0.4 mg to 4 mg CaCO_3_. For both A and B, each calibration group has three technical replicates, and the error bars show one standard deviation of the mean.

To quantify the amount of CO_2_ evolved from the CaCO_3_ standards, the headspace from the H_3_NSO_3_-treated CaCO_3_ was analyzed by GC. A standard curve of CaCO_3_ was prepared with three replicate vials for each input of CaCO_3_ (Fig 1A). Starting at a value of 2.5 mg CaCO_3_, the curve was highly reproducible with an average coefficient of variation (CV) of 0.042% around each CaCO_3_ level. The curve was linear from 2.5 mg to over 80 mg CaCO_3_ with an R^2^ value of 0.998. The limit of detection, defined as the lowest quantity of an analyte that can be detected, was calculated to be 0.32 CCE% [28]. The limit of quantification is the lowest concentration of an analyte that can be determined with acceptable precision and accuracy under standard assay conditions and was calculated to be 0.968 CCE% [28]. To determine if the curve was accurate below 2.5 mg CaCO_3_, dilutions of pure CO_2_ gas were prepared with air as the diluting gas in headspace vials and run on the GC. Again, the standard curve was strongly linear with an R^2^ value of 0.9995 (Fig 1B). We tested values of CO_2_ in the headspace as low as the equivalent to that evolving from the acidification of 0.4 mg CaCO_3_, and even at these low concentrations the method was still very precise, with CVs averaging 0.19% per level.

**Fig 2:**
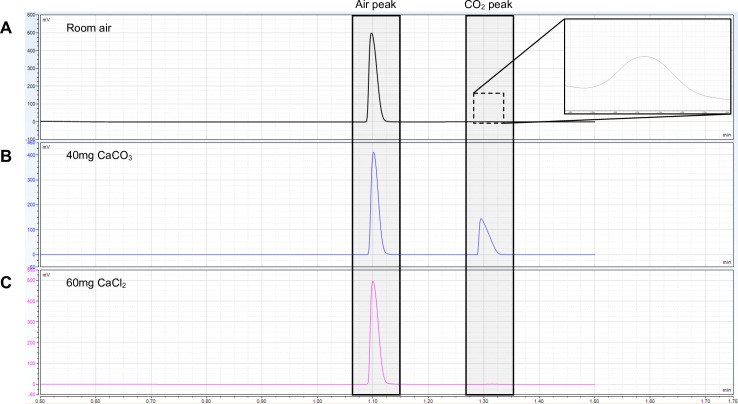
GC chromatograms. (A) Chromatogram showing separation of atmospheric gases into an air peak (composed primarily of N_2_ and O_2_) and a small but detectable CO_2_ peak (see inset). (B) Chromatogram showing separation of gases evolved from the dissolution of 40 mg CaCO_3_ with 5 ml 1.5 M H_3_NSO_3_. (C) Chromatogram resulting from the dissolution of 60 mg calcium chloride with 5 ml 1.5 M H_3_NSO_3_.

The GC method described in this paper allows for the separation and detection of an “room-air” peak, composed primarily of N_2_ and O_2_ that are unresolved by the column, and a CO_2_ peak that elutes later. The CO_2_ peak from laboratory air is small but detectable above the background noise (Fig 2). As noted by Liu et al., the CO_2_ peak area is not linear with increasing CO_2_ headspace concentrations above a relatively low concentration [18]. However, the ratio of the CO_2_ peak area to the air peak area gives a linear curve from the lowest measurable concentrations of CO_2_ to the highest we can maintain without sample leakage from the vials. Therefore, this ratio was used as the y-axis for all plots and standard curves, allowing low and high concentration samples to be measured using the same methodology. To demonstrate the specificity of our system, a vial containing calcium chloride was treated with H_3_NSO_3_ then analyzed by GC; under these conditions, no increase in the background CO₂ peak was observed, suggesting that CO₂ evolution did not occur (Fig 2).

### Calcite crystals dissolve efficiently in HCl and H_3_NSO_3_ but not in H_2_SO_4_

**Fig 3:**
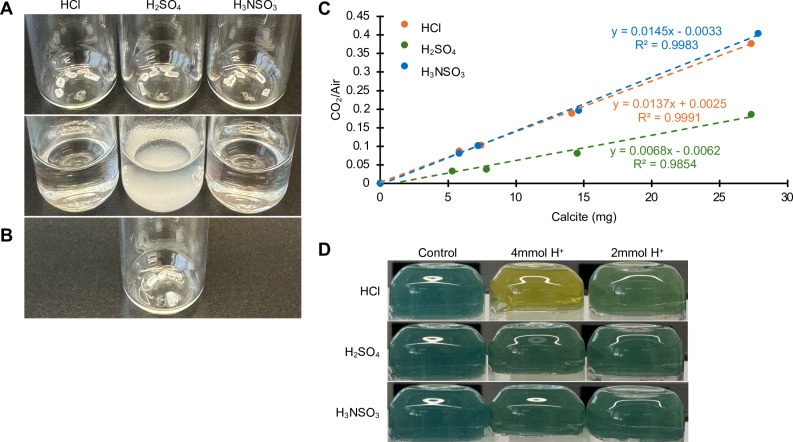
*In vitro* dissolution of calcite. (A) GC vials containing calcite crystals were imaged before and 2 hr after acid treatment. Dissolution of calcite with HCl or H_3_NSO_3_ results in soluble CaCl_2_ (aq) or Ca(H_2_NSO_3_)_2_ (aq), respectively. Dissolution of calcite with H_2_SO_4_ results in insoluble CaSO_4_ (s). (B) Remaining calcite after H_2_SO_4_ and insoluble CaSO_4_ precipitate were removed, revealing the crystals were not fully dissolved by H_2_SO_4_. (C) Results from the GC procedure after 2 h incubation with various acids using calcite instead of CaCO_3_ powder. (D) Test for acidification of headspace by acids in vials (see Materials and Methods for details).


The most common and stable polymorph of mineral CaCO_3_ is calcite, which is naturally heterogeneous in soil, ranging in size from sub-micrometer particles to nodules of 1 cm or greater [11]. To test the efficiency of the acids to dissolve larger CaCO_3_ crystals, we repeated the previous experiments with mineral calcite. Incubation with either HCl or H_3_NSO_3_ resulted in the complete dissolution of calcite and the formation of water-soluble calcium salts (Eq. 1 and 2, and Fig 3A). In contrast, calcite crystals were still present after 1 h incubation with H_2_SO_4_ (Figs 3A and 3B). To observe the remaining calcite crystals, the liquid was carefully aspirated away (Fig 3B). This observation is in line with previous reports suggesting the complete dissolution of calcite is inhibited due to the formation of a calcium sulfate overlayer around the mineral (Eq. 3) [19,20]. We further investigated the effect of these three acids on biologically-produced calcite which was generated and purified from cultures of *Bacillus*. HCl and H_3_NSO_3_ led to the rapid and complete dissolution of biogenic calcite, whereas the mineral was still present after 2 h incubation with H_2_SO_4_ (S1 and S2 Figs Video).

$$CaCO_{3}\left(s\right)+2HCI\left(aq\right)\to CaCl_{2}\left(aq\right)+H_{2}O\left(l\right)+CO_{2}\left(g\right)$$ (1)

$$CaCO_{3}\left(s\right)\;+\;2H_{3}NSO_{3}\left(aq\right)\to Ca{\left(H_{2}NSO_{3}\right)}_{2}\left(aq\right)+H_{2}O\left(l\right)+CO_{2}\left(g\right)$$ (2)

$$CaCO_{3}\left(s\right)\;+\;H_{2}SO_{4}\left(aq\right)\to CaSO_{4}\left(s\right)+H_{2}O\left(l\right)+CO_{2}\left(g\right)$$ (3)

Next, CO_2_ from the sample headspace following calcite dissolution was quantified by GC. When HCl and H_3_NSO_3_ were provided at ~10X molar excess, the calcite readily dissolved and dose-dependent curves were observed (Fig 3C). Addition of H_2_SO_4_ to calcite resulted in the rapid formation of insoluble CaSO_4_ and sampling of the headspace resulted in a heavily deflated curve (approximately half the slope of the curves generated with HCl and H_3_NSO_3_) (Fig 3C). The lower slope in the H_2_SO_4_ curve was consistent with the observation of remaining unreacted calcite crystals (Fig 3B). These data suggest both HCl and H_3_NSO_3_ can liberate CO_2_ from calcite, and the formation of CaSO_4_ inhibits the complete dissolution of calcite in the presence of H_2_SO_4_.

### Sulfamic acid is non-volatile and is compatible with GC

HCl is a volatile acid at room temperature and can result in rapid corrosion of metals. Acid vapors present in the sample headspace can be deleterious toward downstream processes and GC instrumentation. To test for corrosive vapors, headspace pH indicator vials were prepared by adding bromothymol blue to agar and inverting the vial so that the pH indicator agar was at the top of the vial and thus was only exposed to the vapor phase of a liquid present at the bottom of the vial. To test for acid vapors HCl, H_2_SO_4_, and H_3_NSO_3_ were injected into the sealed vials. After three days of incubation the indicator for the HCl-treated samples transitioned from blue to yellow, suggesting the presence of an acidic headspace (Fig 3D). In contrast, the samples treated with H_2_SO_4_ and H_3_NSO_3_ remained blue, and the color was comparable to the negative-control (dH_2_O, no acid).

### H_3_NSO_3_ and HCl sufficiently liberate CO_2_ from agricultural soil carbonates

**Fig 4:**
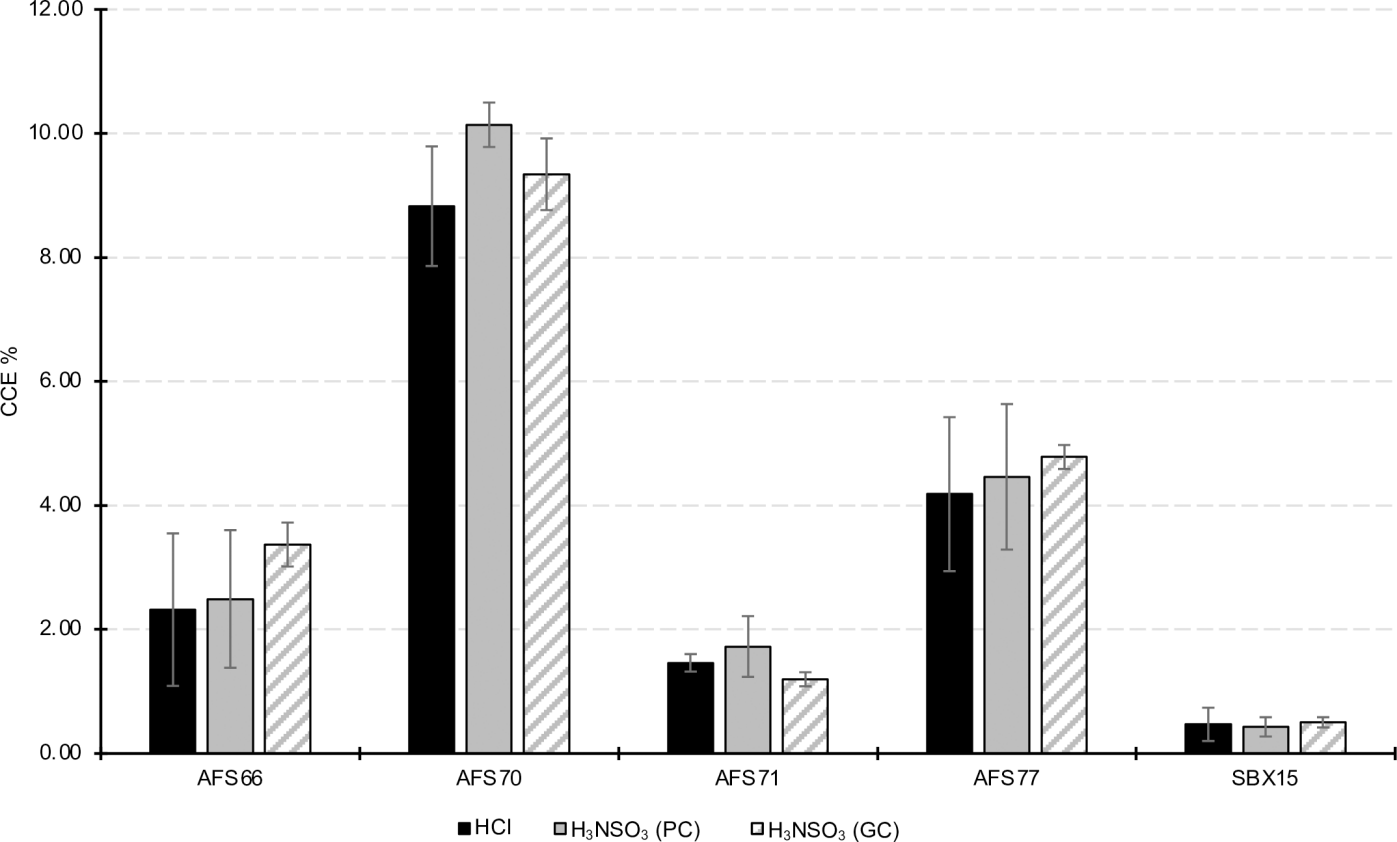
Quantification of soil inorganic carbon in agricultural soil samples. Agricultural soils were prepared as described in Materials and Methods. The three conditions tested were: PC measurements using HCl, PC measurement using H_3_NSO_3_, and GC measurement using H_3_NSO_3_. Three technical replicates were run for each condition, the error bars show one standard deviation of the mean.

HCl is commonly used for carbonate dissolution and CCE% quantification through barometric, gravimetric, and volumetric analyses. However, due to its volatility and corrosive nature, HCl is incompatible with GC instrumentation. To test H_3_NSO_3_ as a suitable alternative to HCl, we first elected to compare the acids using pressure calcimetry because both HCl and H_3_NSO_3_ could be used with this technique interchangeably and, thus, results could be compared directly. Soils were selected from agricultural fields in the US Midwest representing a range of CCE percentages and soil textures (S1 Table). Soil samples treated with either HCl or with H_3_NSO_3_ were statistically equivalent (p < 0.05) in the CCE% values in a TOST test (see Methods) when measured by PC (Fig 4). These data suggest that at equi-volumes and equi-molar amounts of each acid, H_3_NSO_3_ can catalyze the dissolution of carbonates from complex soil matrices on par with HCl.

To demonstrate a proof of concept for measuring soil CCE% by GC, the same panel of agricultural soils was treated with H_3_NSO_3_, and CO_2_ was measured from the sample headspace by GC. The resulting CCE% values were comparable and statistically equivalent (TOST test, p < 0.05) to those obtained from PC (Fig 4). These data suggest H_3_NSO_3_ can catalyze the complete dissolution of carbonates from complex soil matrices comparably to the HCl standard.

**Fig 5:**
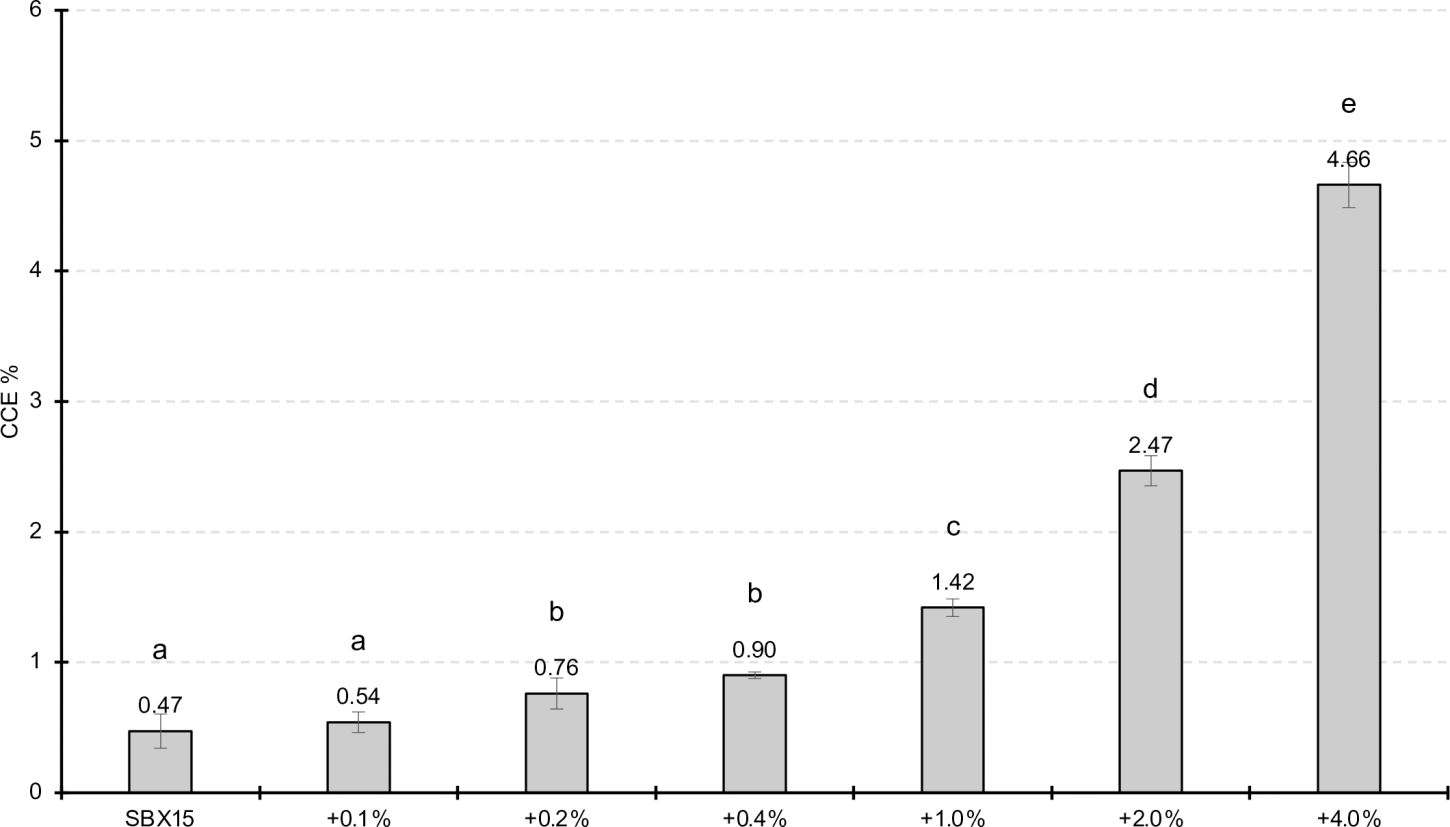
Effect of adding additional CaCO_3_ to soil samples. Additional CaCO_3_ powder was added to soil SBX15 and mixed well. The GC method was performed as described in materials and methods. Three technical replicates were run for each condition and the error bars show one standard deviation of the mean. The alphabetic symbols above the bars represent the groups with significantly different means when compared by Tukey’s all-by-all statistical test (significance level p < 0.05) after testing by ANOVA (p < 0.05).

Finally, we assessed whether the soil matrix itself had an effect on the measured CCE% resulting from sulfamic acid treatment. To test this, increasing amounts of calcium carbonate were mixed with the five agricultural soils tested previously (SBX15, AFS66, AFS70, AFS71, and AFS77). After acidification and GC analysis, the resulting CCE% was proportional to the amount of CaCO_3_ mixed into the sample (Figs 5 and S3). Therefore, we conclude that the matrix itself does not inhibit the dissolution of the soil carbonates using H_3_NSO_3_. Furthermore, this demonstrates the accuracy of the method by correctly measuring the dose-dependent increase in CCE% signal from the added carbonate.

## Discussion

We set out to develop a method to measure soil inorganic carbon that was highly sensitive, precise, and capable of high throughput sample analysis. Gas chromatography provides several advantages over other standard methods to measure SIC such as manometric (e.g., PC), combustion, volumetric and gravimetric methodologies (Table 1). We used PC methods in house to measure SIC and encountered a number of technical and logistical issues that highlighted the advantages of GC. First, a large variety of apparatuses exist for measuring PC, and each has a unique design and sample volume which can affect sensitivity. In our experience, many of these devices are hand-made, potentially leading to poor methodological standardization and ultimately increased levels of error between labs [24,25,29,30]. The same is true for devices that replace the manometer with CO_2_-specific sensors [14]. In contrast, GC has a very standard configuration (10 ml or 20 ml headspace vials) and well-controlled operating conditions that are easily programmable. Third, GC gives an automated measurement with the peak areas assigned by algorithm rather than by a human deciding when the baselines have stabilized therefore reducing human error. GC systems also can be easily outfitted with an autosampler to allow hundreds of samples to be loaded at once, greatly saving operator time.

Another common method for measuring SIC is by combustion. Two main experimental designs are available for this class of SIC measurement. First, a slow ramp method is available to directly measure the soil organic carbon (SOC) at a relatively low temperature followed by a ramp to a higher temperature to measure SIC; the resulting CO_2_ emitted at the different temperatures is measured by IR (e.g., on a LECO RC612). This method gives a simultaneous measure of the various soil carbon components, but with run times between 0.5–1.5 h per sample, the method may lack the sufficient throughput to handle workloads of hundreds to thousands of samples per week.

The second variation for measuring SIC by combustion is to measure each sample twice, measuring total carbon in one measurement and either SOC or SIC in the other measurement, and using subtraction to obtain either SIC or SOC depending on what was not directly measured. In the more common configuration, samples are measured with and without a prior acid treatment to remove the SIC component – and subtracting the two measures gives the SIC value (e.g., on an elemental analyzer). While this method also gives potentially useful information on the SOC component in the sample, the SIC is not directly measured and suffers from increased variability. Error in either of the two measurements that are subtracted to yield the SIC measurement will affect the final SIC measurement. Wagner et al. reported that combustion methods using subtraction have higher CVs than direct measures of SIC at concentrations below 0.5% w/w [29]. Similarly, recent work by Even et al. showed that subtraction methods had higher CVs and methods that directly measure the SIC component had lower CVs [31]. Furthermore, if measurement of SOC is not needed, the combustion method creates additional work and sample tracking as each sample is run on the instrument twice. Acid treatments are performed offline and often in separate containers from the combustion requiring transfer of materials to occur with proper sample tracking and minimal sample loss.

A less common configuration of the combustion method measures total carbon and SIC, with SOC calculated by subtraction. This configuration is supported by specialized instruments such as the PRIMACS MCS made by the company Skalar. Overall, while combustion methods benefit from having standardized equipment, automated measurement, and autosamplers available, in most configurations the increased operational complexity and lack of direct measurement make it less desirable in a high throughput environment for SIC.

A final set of methods to compare the GC method to are the volumetric and gravimetric methods [11,29,32]. In general, these methods are not suitable for high throughput operations as they are manual methods and often require custom equipment in the case of volumetric and are not easily automatable. It is worth noting that while gravimetric methods may not require specialized equipment to perform [32], they provide nonetheless an indirect measure of inorganic carbon and has been reported to suffer from low sensitivity and high CVs at low % SIC [25].

In comparing the costs of the GC method to the other methods, it is helpful to consider the cost of the instrument as well as the cost of materials and consumables (e.g., vials and caps). A GC with an autosampler is a significant expense for a laboratory. But considering the multiple applications throughout an analytical laboratory, the cost of the GC instrument may be partially offset. The cost of the GC is equivalent to combustion instruments, but significantly higher than instruments required for PC, volumetric, and gravimetric methods. The materials and consumables cost for the GC method is approximately $1 USD per sample including the vial, cap, and acid reagents. The consumables are generally disposable for the GC method. The combustion method has crucibles that can be reused approximately several times according to the manufacturer, but they are proportionally more expensive, and therefore make the consumables costs comparable for these methods. But given that most higher throughput combustion methods require measuring the soil sample twice for SIC, the GC method may be significantly cheaper in the end. Consumables for PC could be disposable as for GC, depending on whether the instrument is custom made and has a built-in reaction chamber, or the consumables could be cleaned and reused. Volumetric and gravimetric methods generally have reusable consumables but are also not amenable to high throughput operation. There is a significant cost in terms of time and effort to clean hundreds of vials a day as well as a significant space requirement to store them while cleaning and drying. Overall, the ability to use standard and cheap consumables that are available from multiple suppliers is a strong feature of the GC method enabling high throughput operation.

**Table 1:** Comparison of available measurement options for SIC.

	GC	Combustion	PC	Volumetric	Gravimetric
**Direct measure of CO**_**2**_	Yes	Yes^a^	No	No	No
**Standardized equipment**	Yes	Yes	No	No	Yes^b^
**Automated measurement**	Yes	Yes	No	No	No
**Autosampler**	Yes	Yes	No	No	No

^a^Direct measure of CO_2_ available in some configurations. TC and SOC available if needed.

^b^No specialized equipment required

This manuscript describes the first use of GC for direct measurement of inorganic carbonates in soil. Others have reported using GC to measure carbonates from various environmental samples, but for various reasons these methods are not directly useful for SIC. For example, Dai et al. developed a GC method to measure carbonate content in paper pulp using sulfuric acid [17]. While choice of this acid may be acceptable in this context, we demonstrate that sulfuric acid should be avoided in measurement of SIC as it does not sufficiently dissolve soil carbonates, presumably due to the size of the carbonate crystals present. Other examples of using GC for carbonate measurement have been reported in the literature but use hydrochloric acid. These include methods to measure carbonates in barite ore [18], and methods to measure bicarbonate and dissolved CO_2_ in water samples [16]. We demonstrate the headspace volatility of HCl in our assay conditions and advise against its use with GC as corrosive materials are not compatible with the long-term maintenance of the instrument.

Based on the results of this study, we see the GC instrument as a key part of a larger laboratory automation system to enable vastly expanded measurement of SIC. GC instruments are widely used in academic and industrial laboratories, making service and maintenance on these instruments straight forward to schedule through original and third-party vendors. This contrasts to specialized carbon measurement instruments where service technicians may not be immediately available to fix the instrument. The use of automated syringe pumps can facilitate acid injection for carbonate dissolution, and GC autosamplers free up the end user’s time. Automated scripts for data analysis and data quality control can aid processing the large amount of collected data. Together with a laboratory that is set up for higher throughput experimentation, this method allows for SIC measurement not just at a greater scale but also at higher resolution by sampling more frequently in fields.

In conclusion, a novel GC method for the measurement of SIC was demonstrated in this study. We identify sulfamic acid as a strong acid to give complete dissolution of the carbonates while also being non-volatile. We provide evidence for the robust performance of this method in a variety of soils and discuss the merits of the method relative to other available methods for SIC measurement, including PC, combustion, gravimetric and volumetric methods. Overall, the results of our GC method suggest that it fills an open niche for SIC measurement, offering an operationally simple, high throughput, precise, and sensitive approach when SIC measurement is needed.

## Supporting information

S1 Fig *In vitro* dissolution of biogenic calcite. (A) 22 ml GC vials were prepared with 10 mg biogenic calcite. (B) The samples were injected with 4 ml acid (equivalent to>40X molar excess acid) and incubated at room temperature for 2 h. Dissolution of calcite with HCl or H_3_NSO_3_ results in soluble CaCl_2_ (aq) or Ca(H_2_NSO_3_)_2_ (aq) respectively. Dissolution of calcite with H_2_SO_4_ results in insoluble CaSO_4_ (s).
(TIF)

S2 Fig *In vitro* dissolution of individual biogenic calcite crystals. Four individual crystals were placed into separate wells on a glass microscope slide. The crystal in the upper left is untreated for comparison. A drop of 2 M H_2_SO_4_ was dispensed onto the crystal in the lower left, resulting in effervescence observed as bubbles released from the crystal. Next, 1 M H_3_NSO_3_ was dispensed onto the crystal in the lower right, followed by 1 M HCl in the upper right hand corner. H_3_NSO_3_ and HCl resulted in the complete dissolution of the calcite crystals, evident by effervescence and the disappearance of the crystals.
(MOV)

S3 Fig Effect of adding additional CaCO_3_ to soil samples. Additional CaCO_3_ (0.4% or 4%, w/w) was added to agricultural soils AFS66, AFS70, AFS71, and AFS77, and mixed well. 0.5 g of this mixture was added to 22 ml GC vials, capped, and injected with 5 ml 1.5 M H_3_NSO_3_. Three technical replicates were run for each condition and the error bars show one standard deviation of the mean. Letters (a, b or c) above each bar indicate statistically significant differences among the treatments within each group of soil. For the AFS66, AFS70, and AFS77 groups, one-way analysis of variance (ANOVA), p-value < 0.01 and Tukey’s multiple comparison test, p-value < 0.05 was used. For the AFS71 group: due to lack of homogeneity of variance, Brown-Forsythe and Welch ANOVA test, p-value < 0.0001 and Dunnett’s multiple comparisons test, p-value < 0.05 was used.
(TIF)

S1 Table Soil texture data for soils tested in this study. (PDF)

S2 Table Raw data for figures presented in this study. (XLSX)
