# Supplementary figures and images for: Quantification of soil inorganic carbon using sulfamic acid and gas chromatography
Source: PLoS One. 2025 May 5;20(5):e0320778. doi: 10.1371/journal.pone.0320778 (PMC12052155; doi:10.1371/journal.pone.0320778)

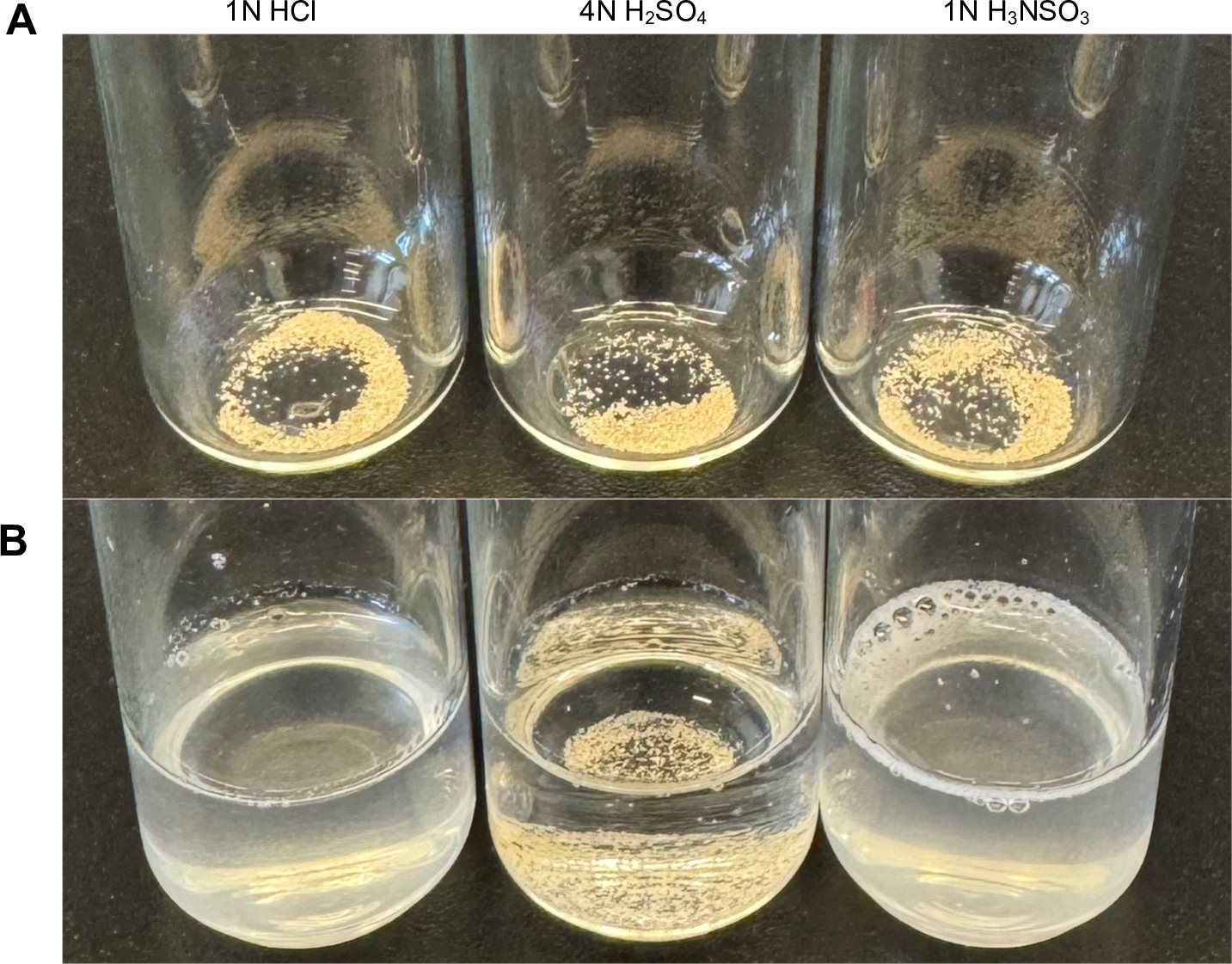

Supplement: S1 Fig — (A) 22 ml GC vials were prepared with 10 mg biogenic calcite. (B) The samples were injected with 4 ml acid (equivalent to>40X molar excess acid) and incubated at room temperature for 2 h. Dissolution of calcite with HCl or H3NSO3 results in soluble CaCl2 (aq) or Ca(H2NSO3)2 (aq) respectively. Dissolution of calcite with H2SO4 results in insoluble CaSO4 (s). (TIF) [file pone.0320778.s001.tif]

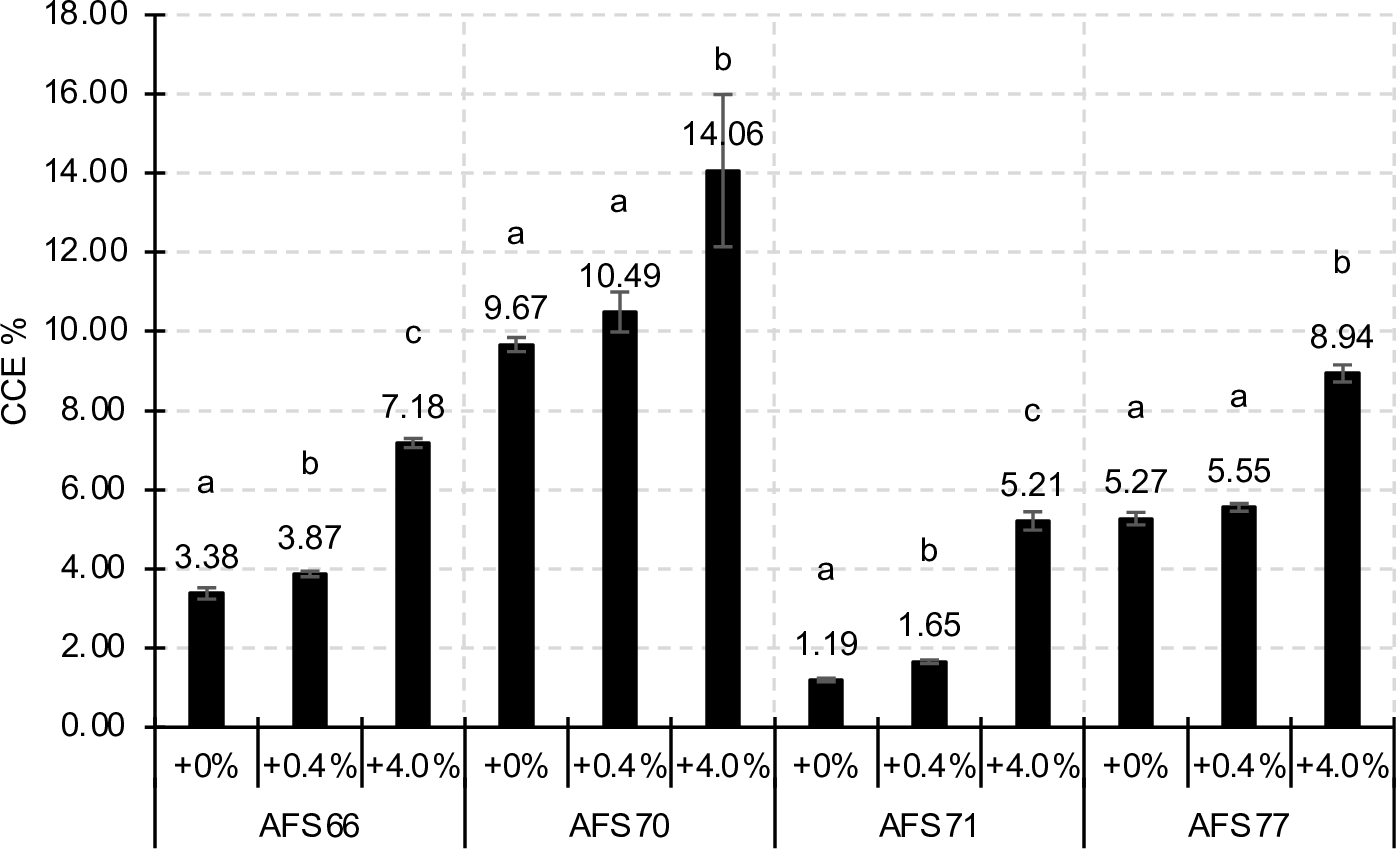

Supplement: S3 Fig — Additional CaCO3 (0.4% or 4%, w/w) was added to agricultural soils AFS66, AFS70, AFS71, and AFS77, and mixed well. 0.5 g of this mixture was added to 22 ml GC vials, capped, and injected with 5 ml 1.5 M H3NSO3. Three technical replicates were run for each condition and the error bars show one standard deviation of the mean. Letters (a, b or c) above each bar indicate statistically significant differences among the treatments within each group of soil. For the AFS66, AFS70, and AFS77 groups, one-way analysis of variance (ANOVA), p-value < 0.01 and Tukey’s multiple comparison test, p-value < 0.05 was used. For the AFS71 group: due to lack of homogeneity of variance, Brown-Forsythe and Welch ANOVA test, p-value < 0.0001 and Dunnett’s multiple comparisons test, p-value < 0.05 was used. (TIF) [file pone.0320778.s003.tif]
