# Supplementary material for: Quantification of soil inorganic carbon using sulfamic acid and gas chromatography
Source: PLoS One. 2025 May 5;20(5):e0320778. doi: 10.1371/journal.pone.0320778 (PMC12052155; doi:10.1371/journal.pone.0320778)
Supplement: S1 Table — (PDF) [file pone.0320778.s004.pdf]

Soil ID	% Sand	% Silt	% Clay	Soil Texture
AFS66	17.6	49.4	33	Silty clay loam
AFS70	40	37	23	Loam
AFS71	40	37	23	Loam
AFS77	5	47	48	Silty clay
SBX15	87	9	4	Sand
